# Evaluation of trends in hospital antimicrobial use in the Lao PDR using repeated point-prevalence surveys-evidence to improve treatment guideline use

**DOI:** 10.1016/j.lanwpc.2022.100531

**Published:** 2022-07-09

**Authors:** Vilada Chansamouth, Danoy Chommanam, Tamalee Roberts, Sommay Keomany, Viladeth Paphasiri, Chanthala Phamisith, Siho Sengsavang, Khamsay Detleuxay, Phisith Phoutsavath, Sengvong Bouthavong, Anousone Douangnouvong, Manivanh Vongsouvath, Sommana Rattana, Bounxou Keohavong, Nicholas P.J. Day, Paul Turner, H. Rogier van Doorn, Mayfong Mayxay, Elizabeth A. Ashley, Paul N. Newton

**Affiliations:** a Lao-Oxford-Mahosot Hospital-Wellcome Trust Research Unit (LOMWRU), Mahosot Hospital, Vientiane City, Lao PDR; b Microbiology Laboratory, Mahosot Hospital, Vientiane City, Lao PDR; c Centre for Tropical Medicine & Global Health, Nuffield Department of Medicine, University of Oxford, Oxford, England, UK; d Salavan Provincial Hospital, Salavan Province, Lao PDR; e Xiengkhuang Provincial Hospital, Xiengkhuang Province, Lao PDR; f Savannakhet Provincial Hospital, Savannakhet Province, Lao PDR; g Vientiane Provincial Hospital, Vientiane Province, Lao PDR; h Mahosot Hospital, Vientiane City, Lao PDR; i Luang Namtha Provincial Hospital, Luang Namtha Province, Lao PDR; j Department of Healthcare and Rehabilitation, Ministry of Health, Vientiane City, Lao PDR; k Department of Food and Drug, Ministry of Health, Vientiane City, Lao PDR; l Institute of Research and Education Development (IRED), University of Health Sciences, Ministry of Health, Vientiane Lao PDR; m Mahidol Oxford Tropical Medicine Research Unit (MORU), Faculty of Tropical Medicine, Mahidol University, Bangkok, Thailand; n Cambodia Oxford Medical Research Unit, Siem Reap, Cambodia; o Oxford University Clinical Research Unit, Hanoi, Viet Nam; p Faculty of Infectious and Tropical Diseases, London School of Hygiene and Tropical Medicine, London, UK

**Keywords:** Antimicrobial use, Prescription, Hospital, Laos, Antimicrobial resistance, Point prevalence survey

## Abstract

**Background:**

Antimicrobial use (AMU) is a key driver of antimicrobial resistance (AMR). There are few data on AMU, to inform optimizing antibiotic stewardship, in the Lao PDR (Laos).

**Methods:**

Point prevalence surveys (PPS) of AMU were conducted at four-month intervals in six general hospitals across Laos from 2017 to 2020, using modified Global-PPS data collection tools. The surveys focused on AMU amongst hospitalized inpatients.

**Findings:**

The overall prevalence of inpatient AMU was 71% (4,377/6,188), varying by hospital and survey round from 50·4% (135/268) to 88·4% (61/69). Of 4,377 patients, 44% received >one antimicrobial. The total number of prescriptions assessed was 6,555. Ceftriaxone was the most commonly used (39·6%) antimicrobial, followed by metronidazole (17%) and gentamicin (10%). Pneumonia was the most common diagnosis among those prescribed antimicrobials in both children aged ≤5 years (29% among aged ≤1 year and 27% among aged >1 to ≤5years) and adults aged ≥15 years at 9%. The percentage of antimicrobial use compliant with local treatment guidelines was 26%; inappropriate use was mainly found for surgical prophylaxis (99%). Adult patients received ACCESS group antimicrobials less commonly than children (47% vs 63%, p-value<0·0001). Most WATCH group prescriptions (99%) were without a microbiological indication.

**Interpretation:**

AMU among hospitalized patients in Laos is high with frequent inappropriate use of antimicrobials, especially as surgical prophylaxis. Continued monitoring and enhanced antimicrobial stewardship interventions are needed in Lao hospitals.

**Funding:**

The Wellcome Trust [Grant numbers 220211/Z/20/Z and 214207/Z/18/Z] and bioMérieux.

**Research in context**

**Evidence before this study**

• Antimicrobial use (AMU) is one of the key drivers of antimicrobial resistance (AMR).
• Surveillance and monitoring of AMU and AMR are conducted in many countries to inform AMR control strategies.
• Data on hospital antimicrobial use in the Lao PDR (Laos) are scarce.

**Added value of this study**

• Antimicrobial use in hospitalized patients in Laos is higher than surrounding countries and most regions across the globe.
• Cephalosporins and penicillins were the most commonly used antimicrobials in all age groups; while aminoglycoside use was more common among children aged ≤5 years and nitroimidazoles were more commonly prescribed in older children and adults. Overall use of ACCESS group antimicrobials was 50% in Laos which was less than the WHO target of 60% and higher in children than adults.
• Pneumonia was the number one indication for antimicrobial prescription in both children and adults.
• Appropriate use of antimicrobials in hospitals in Laos according to prescribing indication was only 26%. Inappropriate use of antimicrobials was mainly explained by use for prophylaxis.

**Implications of all the available evidence**

• These data suggest targets for appropriate solutions to improve antimicrobial use to control AMR in Laos.
• Appropriate antimicrobial stewardship programs, including comprehensive antimicrobial use guidelines and regular monitoring of AMU should be applied to the whole country.

Alt-text: Unlabelled box

## Introduction

Acquired antimicrobial resistance (AMR) emerges quickly in some pathogens soon after the deployment of many antimicrobials, reducing their efficacy^1^^,^^2^ and has become an urgent global health concern, causing increasing mortality and healthcare costs, especially in low and middle-income countries.^3^, ^4^, ^5^ There is demand for new classes of antibiotics, as well as to reconsider old antibiotics which are active against multidrug resistant bacteria.^6^ Although AMR may be intrinsic, antimicrobial use (either appropriate or inappropriate) is one of the key drivers of acquired AMR.^7^ Inpatient antimicrobial prescription data from the Global Point Prevalence Survey (Global-PPS) conducted in 53 countries worldwide (64% of participating hospitals were from European countries) in 2015 showed that the proportion of hospitalized patients prescribed antimicrobials ranged from 27% to 50%. Some 77% of 36,792 antibiotic prescriptions were compliant with local guidelines.^8^ A multicentre study of hospital antimicrobial use in 2019 in four African countries (Ghana, Uganda, Zambia and Tanzania) showed a similar pattern of hospital antimicrobial use which ranged from 30% to 57%. Compliance to local guidelines ranged from 55%-88%.^9^ Studies from WHO Western Pacific countries, such as Malaysia, Japan, China and Vietnam, have reported the use of antimicrobials in hospitals ranging from 28.5% to 67.4%.^10^, ^11^, ^12^, ^13^ Understanding hospital antimicrobial usage within countries may help to find effective tools to combat AMR tailored to the local context. A recent situational analysis of AMR and AMU in humans, animals and the environment in Lao PDR (Laos) found that data on AMR and antimicrobial use (AMU) in Laos were sparse. Use of antimicrobials in inpatients ranged from 45 to 70% between 2004 and 2018. Appropriateness of use has not been assessed before.^14^ Laos began participating in the Global-PPS of antimicrobial consumption and resistance in 2017 to provide the first data on hospital antimicrobial prescribing in the country as a part of a project on surveillance of antimicrobial use and resistance in Laos (https://livedataoxford.shinyapps.io/amulaos/). The aim of this study was to describe the patterns of hospital antimicrobial use in six general hospitals across Laos over a 4-year period, from 2017 to 2020, as a basis for informing AMR policy, action and implementation in Laos.

## Methods

### Study design and setting

Point prevalence surveys (PPS) of antimicrobial use were repeatedly conducted in inpatients in six hospitals: two referral (200-450 beds, including Mahosot Hospital in Vientiane Capital and Savannakhet Provincial Hospital) and four provincial hospitals (60–100 beds), including Luang Namtha, Xiengkhuang, Vientiane and Salavan Provincial Hospitals. Mahosot, Xiengkhuang, Luang Namtha and Salavan participated in the surveys from mid-2017, with Vientiane Provincial Hospital joining in 2018 and Savannakhet in 2019. These six hospitals were included in the survey because they were participating in aetiology of fever studies in Laos and because they were geographically dispersed, with two hospitals located in the north, two in the centre, and two in the south of Laos. These six hospitals in one prefecture and five out of 17 provinces were considered representative of different regions in Laos (Supplementary Figure 1).

### Data collection

Data collection was performed using an adapted version of the Global-PPS protocol (2019)^15^ (Lao data were submitted to Global-PPS until the end of 2018). Inpatient records from all wards recorded as present at 8:00 am on the survey day were screened and antimicrobial prescriptions recorded; all screened patients were counted as the denominator. Survey days were a convenience sample of weekdays, one day per ward, three times a year. Unlike the Global-PPS protocol, healthy newborn babies on maternity wards were not included in the denominator. Data on topical antimicrobials were not collected. Surveys were not conducted during public holidays or weekends. All patients documented with a prescription of any kind of antimicrobial agent(s) prescribed in the 24 h before 8:00 am of the survey day were counted as the numerator. Patients’ demographic and clinical characteristics and treatment status, including age, body weight, ward, antimicrobial agent(s), route of administration, dosage, unit, diagnoses, and prescribing indication were recorded. Data were collected directly from hospital charts, without interviewing patients or ward staff, unless the number of charts and number of patients were inconsistent or handwriting was illegible. Each ward survey was completed within the survey day.

PPS were conducted three times a year from July 2017 to December 2020. The survey rounds were based on Lao seasons with the wet season from May to October and dry season from November to April.^16^ The dry season consists of cool dry (November to February) and hot dry (March to April) seasons. The first survey round of the year was in the hot dry season (March-April ± one month), the second survey round was in the wet season (May-October ± one month) and the third survey round was in the cool dry season (November-February ± one month). Prescribing indications were classified into treatment of infection, medical prophylaxis (medical prophylaxis-general, medical prophylaxis-trauma and medical prophylaxis-vaginal delivery), surgical prophylaxis, unclear and unknown (Supplementary Table 1).

Spectrum (broad or narrow) of antibiotics was classified based on Hagedoorn *et al*.^17^ Treatment was classified as empiric or targeted (i.e. based on microbiology results). Compliance with the guidelines was assessed using local treatment guidelines at the time of prescribing (pre-2021 guidelines). Compliance with the guidelines was evaluated according to the diagnosis in the patient's medical notes at the start of the antimicrobial treatment and for diagnoses which were available in the local treatment guidelines. Compliance to guidelines was not assessed when there were no local guidelines or the prescription indications were unknown or unclear. Guideline compliance and appropriateness in this study were based solely on the choice of antimicrobial(s), regardless of the dosage, route of administration and duration of use. Antimicrobial prescriptions were also reassessed retrospectively using new local antimicrobial prescribing guidelines which were released in January 2021 (2021-guidelines) (Supplementary Table 2)^18^^,^^19^ to compare the appropriateness of the use of antimicrobials if these detailed antimicrobial prescribing guidelines had been available at the time of prescribing.

### Data analysis

Descriptive analysis was performed for patients’ characteristics, the trend of antimicrobial prescriptions through seasons, survey round, and antimicrobial prescriptions by medical specialty and organ involved. Frequency, mean (95% confidence interval (CI)), and median with interquartile range (IQR) were used to describe data. Antimicrobial prescriptions were described based on the prescribing indications, WHO AWaRe classification (ACCESS, WATCH, RESERVE or Not recommended)^20^ and whether the prescription adhered to the local guidelines. Pearson's chi-squared test or Fisher's exact test were used to compare the proportions of categorical variables. Time series data analysis was used to assess the seasonality and trends of antimicrobial use over the survey period using Chi-squared goodness of fit test and binary regression.

### Ethics approval and patient consent

Monitoring AMU was introduced as a routine surveillance activity in Laos and no patient identifiable data were collected. Surveys were conducted with the permission of the participating hospitals and the Lao Ministry of Health and were not subject to ethical review.

### Role of the funding source

This work was funded by the Wellcome Trust [Grant numbers 220211/Z/20/Z and 214207/Z/18/Z] and bioMérieux – Singapore who provided a startup grant for the project. The funders of this study had no role in the study design, data collection, analysis, interpretation or writing the report. For the purpose of open access, the author has applied a CC BY public copyright licence to any Author Accepted manuscript version arising from this submission.

## Results

**Figure 1:**
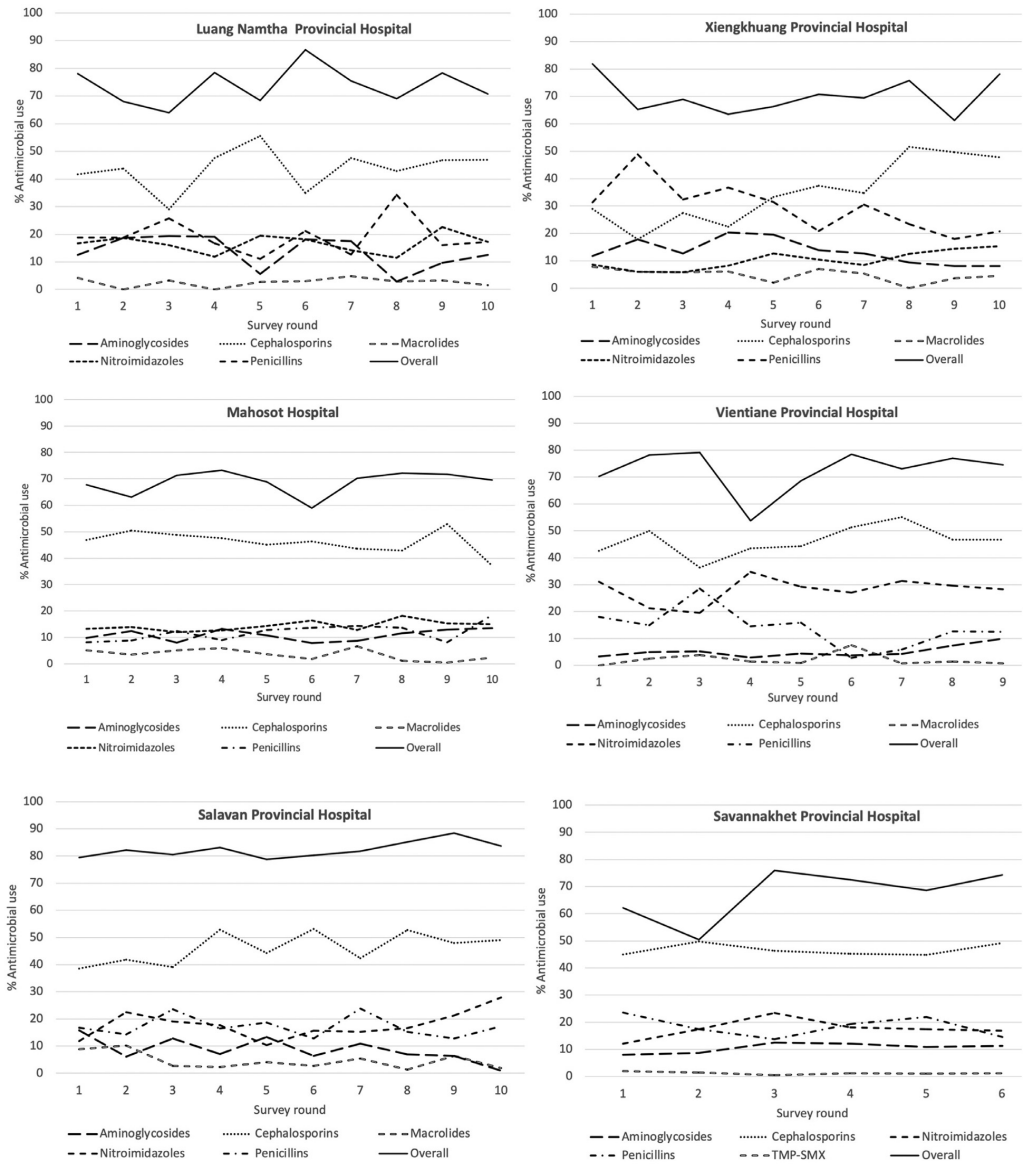
Antimicrobial use in six survey hospitals in Laos, from 2017 to 2020, for the five most commonly used antimicrobials.

Between July 2017 and December 2020 (41 months), ten rounds of antimicrobial use PPS were conducted in six hospitals across Laos (Supplementary Figure 1). Over these 41 months, records of 6,188 inpatients were screened for antimicrobial use; revealing that 4,377 (71% (69% - 72%, 95% CI)) inpatients received antimicrobial treatment, with 6,555 antimicrobial prescriptions in total. The use of antimicrobials varied from the lowest prevalence of 50·4% (135/268) in one survey round in Savannakhet Provincial Hospital to the highest of 88·4% (61/69) in Salavan Provincial Hospital (Figure 1). The majority of patients included were adults (≥15 years), 3421/4377 (78%). Infants (≤1 year) accounted for 435/4,377 (10%). Of 435 infants, 217 (50%) were neonates (≤28 days). Of the 4,377 patients, 1,336/4,377 (30%) were admitted to surgical departments and 44% (1,918/4,377) were prescribed more than one antimicrobial (Table 1).

**Table 1:** Patient characteristics and antimicrobial use in six survey hospitals across Laos from 2017 to 2020.

Characteristic/Year	2017	2018	2019	2020	Total
**Number of survey rounds**	1	3	3	3	10
**Number of survey hospitals**	4	5	6	6	6
**Screened patient documents**^a^	489	1,450	2,161	2,088	6,188
**Patients with antimicrobial(s) prescribed - n (%)**	360 (74)	1,036 (71)	1,449 (67)	1,532 (73)	4,377 (71)
**Number of prescriptions**	533	1,518	2,187	2,317	6,555
**Antimicrobial use by hospital – n (%)**					
Mahosot Hospital	176/260 (68)	426/618 (69)	412/631 (65)	401/564 (71)	1,415/2073 (68)
Vientiane Provincial Hospital	-	154/202 (76)	175/261 (67)	223/298 (75)	552/761 (72·5)
Luang Namtha Provincial Hospital	32/41 (78)	72/102 (81)	102/132 (77)	124/169 (73)	330/444 (74)
Xiengkhuang Provincial Hospital	90/110 (81·8)	199/302 (66)	215/312 (69)	202/287 (70)	706/1,011 (70)
Salavan Provincial Hospital	62/78 (79·5)	185/226 (82)	198/247 (80)	179/209 (86)	624/760 (82)
Savannakhet Provincial Hospital	-	-	347/578 (60)	403/561 (72)	750/1,139 (66)
**Age, median (IQR) years**	33 (17-57)	32 (18-54)	31 (17-54)	32 (18-52)	32 (18-54)
**Age category - n (%)**^a^ ≤ 1 year	31 (9)	104 (10)	138 (9)	162 (11)	435 (10)
> 1 to ≤ 5 years	31 (9)	64 (6)	104 (7)	90 (6)	289 (7)
> 5 to ≤ 14 years	19 (5)	51 (5)	80 (6)	81 (5)	232 (5)
≥ 15 years	279 (77)	816 (79)	1,127 (78)	1,199 (78)	3,421 (78)
**Department - n (%)**^a^					
Intensive Care	16 (4)	79 (8)	104 (7)	136 (9)	335 (8)
Medicine	108 (30)	270 (26)	331 (23)	345 (22)	1,054 (24)
Paediatrics	49 (14)	122 (12)	221 (15)	201 (13)	593 (14)
Surgery	120 (33)	307 (30)	449 (31)	454 (30)	1,330 (30)
Obstetrics & Gynaecology	50 (14)	199 (19)	246 (17)	271 (18)	766 (17)
Other	17 (5)	59 (6)	98 (7)	125 (8)	299 (7)
**Number of antimicrobial(s) – n (%)**^a^ One	210 (58)	608 (59)	789 (54)	852 (56)	2,459 (56)
Two	127 (35)	377 (36)	586 (40)	578 (38)	1,668 (38)
≥ Three	23 (6)	51 (5)	74 (5)	102 (7)	250 (6)
**Route of administration – n (%)**^b^ Oral	140 (26)	353 (23)	392 (18)	340 (15)	1,225 (19)
Parenteral	393 (74)	1,165 (77)	1,795 (82)	1,977 (85)	5,330 (81)
**Indication - n (%)**^b^					
Treatment of infection	295 (55)	798 (53)	1,173 (54)	1,198 (52)	3,464 (53)
Medical prophylaxis-general	36 (7)	81 (5)	120 (5)	136 (6)	373 (6)
Medical prophylaxis-trauma	6 (1)	21 (1)	51 (2)	78 (3)	156 (2)
Medical prophylaxis-vaginal delivery	22 (4)	97 (6)	114 (5)	124 (5)	357 (5)
Surgical prophylaxis	125 (23)	366 (24)	453 (21)	480 (21)	1,424 (22)
Unclear indication	12 (2)	34 (2)	56 (3)	82 (4)	184 (3)
Unknown	37 (7)	121 (8)	220 (10)	219 (9)	597 (9)
**Indication documented in patient records – n (%)**^b^	482 (90)	1,356 (89)	1,900 (87)	2,014 (87)	5,752 (88)
**Treatment – n (%)**^b^ Empirical	521 (98)	1,491 (98)	2,167 (99)	2,288 (99)	6,467 (99)
Targeted	12 (2)	27 (2)	20 (1)	29 (1)	88 (1)
**Stop/review date documented - n (%)**^b^	4 (0·7)	28 (1·8)	4 (0·2)	16 (0·7)	52 (0·8)
**Targeted sites for prescriptions - n (%)**^b^					
Bone and Joint	8 (1)	44 (3)	67 (3)	61 (3)	180 (3)
Cardiovascular System	2 (0·4)	8 (0·5)	12 (0·6)	13 (0·6)	35 (0·5)
Central Nervous System	15 (3)	24 (2)	46 (2)	89 (4)	174 (3)
Ear-Nose-Throat	41 (8)	86 (6)	149 (7)	91 (4)	367 (6)
Gastrointestinal	133 (25)	362 (24)	583 (27)	556 (24)	1,634 (25)
Male genitalia	10 (2)	26 (2)	21 (1)	34 (1)	91 (1)
Obstetrics/Gynaecology	75 (14)	307 (20)	396 (18)	478 (21)	1,256 (19)
Ophthalmology	4 (0·7)	9 (0·6)	9 (0·4)	12 (0·5)	34 (0·5)
Respiratory tract	105 (20)	301 (20)	318 (15)	286 (12)	1,010 (15)
Sepsis	26 (5)	40 (3)	104 (5)	171 (7)	341 (5)
Skin and Soft Tissue	39 (7)	118 (8)	159 (7)	219 (9)	535 (8)
Urinary tract	45 (8)	101 (7)	175 (8)	132 (6)	453 (7)
Not defined	30 (6)	92 (6)	148 (7)	175 (7)	445 (7)
**Compliance with pre-2021 guidelines – n (%)**^b^					
Compliant	122 (32)	264 (26)	353 (24)	397 (26)	1,136 (26)
Not compliant	263 (68)	766 (74)	1,115 (76)	1,117 (74)	3,261 (74)
Not assessed (%)	148 (28)	488 (32)	719 (33)	803 (35)	2,158 (33)
**Compliance with 2021-guidelines – n (%)**^b^					
Compliant	172 (37)	449 (34)	672 (36)	671 (34)	1,964 (35)
Not compliant	287 (63)	869 (66)	1,210 (64)	1,307 (66)	3,673 (65)
Not assessed (%)	93 (17)	300 (20)	480 (22)	553 (24)	1,426 (22)

a Denominator was number of patients with antimicrobial(s).

b Denominator was number of prescriptions.

### Trends of antimicrobial use

**Figure 2:**
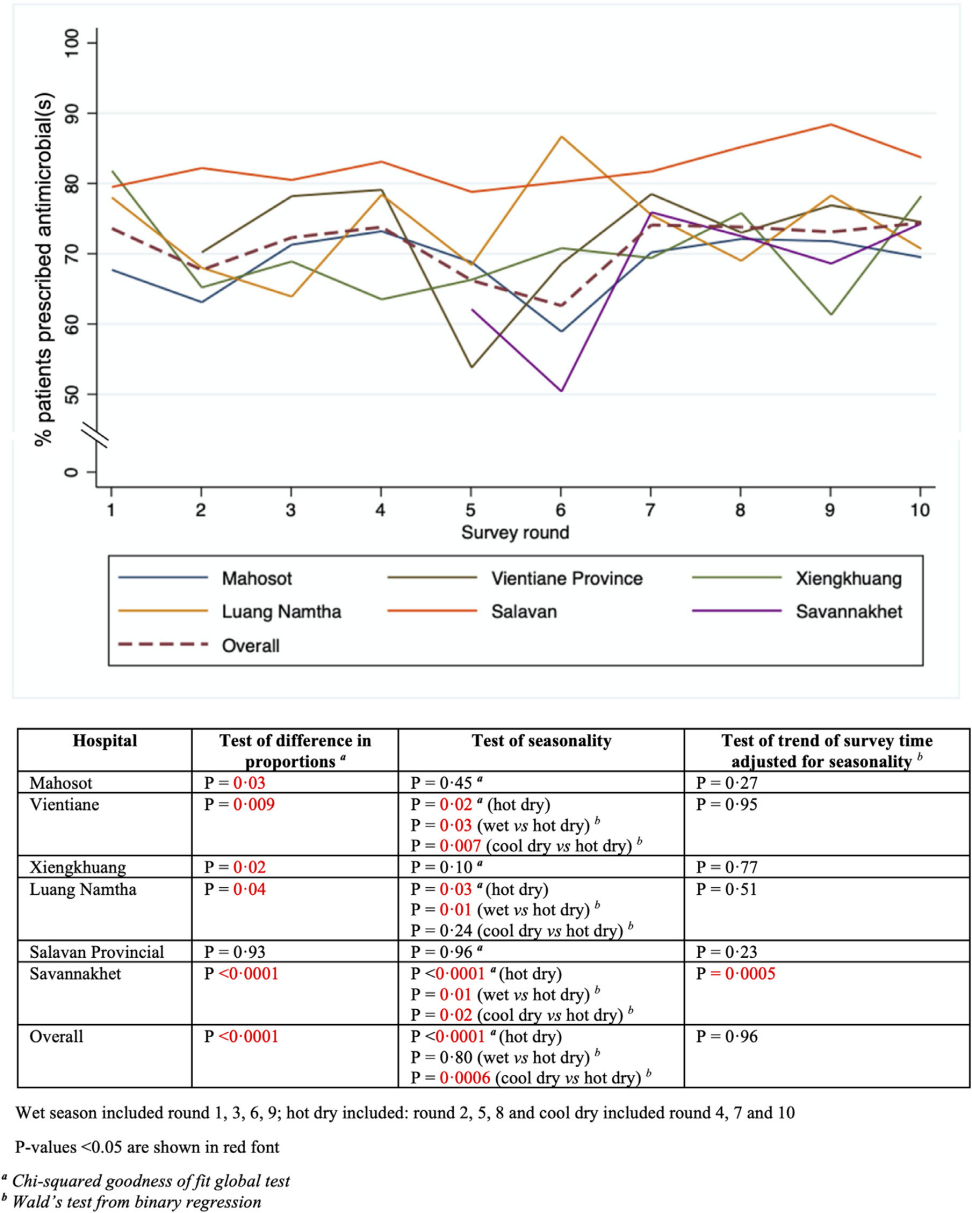
Trends of antimicrobial use in six survey hospitals in Laos from 2017 to 2020.

Overall antimicrobial use varied between hospitals over ten survey rounds. The highest use (means (95% CI) was found at Salavan Provincial Hospital at 82·3% (80·2%-84·4%), followed by Luang Namtha at 74·8% (68·8%-80·9%), Vientiane Province at 72·5% (66·4%-78·6%), Xiengkhuang at 70% (65·2%-74·8%), Mahosot at 68·6% (65·4%-71·8%-77·4%). There were significant differences in the proportion of patients prescribed antimicrobials in each survey round by individual hospital (Mahosot: *p* = 0·03; Vientiane Province: *p =* 0·009; Xiengkhuang: *p =* 0·002; Luang Namtha: *p =* 0·04 and Savannakhet: *p* < 0·0001, except Salavan: *p =* 0·93). Season had no discernable effect on antimicrobial use at Mahosot Hospital (*p =* 0·45), Xiengkhuang (*p =* 0·10) and Salavan (*p =* 0·96). However, the use of antimicrobials varied by seasons in Vientiane Province, Luang Namtha and Savannakhet (Figure 2). The overall proportions of the use of each antimicrobial class did not change by season over each survey year (Supplementary Table 3), apart from the use of macrolides in 2019 which was higher in the cool dry season (34 (4·7%)) but with similar proportions for the hot dry (18 (2·8%)) and wet seasons (19 (2·3%)) with *p =* 0·03 (Supplementary Table 3). After controlling for seasonality, there was no trend in the proportion of patients prescribed antimicrobials over the survey period in each survey hospital (Mahosot Hospital: *p =* 0·27, Vientiane Province: *p =* 0·95, Xiengkhuang: *p =* 0·77, Luang Namtha: *p =* 0·51 and Salavan: *p =* 0·23); except Savannakhet: *p =* 0·0005 (Figure 2).

### Choice of antimicrobials

Among 6,555 antimicrobial prescriptions from 2017 to 2020, antibiotics accounted for 6,433/6,555 (98%), anti-tuberculosis drugs for 49/6,555 (0·8%), antifungals for 47/6,555 (0·7%), antivirals (aciclovir and antiretrovirals) for 13/6,555 (0·2%), anti-helminthics for 12 (0·2%) and an antimalarial for 1 (0·02%). Of all prescriptions, the cephalosporins class was the most prescribed at 2,919/6,555 (44·5%), followed by penicillins at 1,116/6,555 (17%), then nitroimidazoles at 1,104/6,555 (16·8%), aminoglycosides at 679/6,555 (10·4%) and macrolides at 213/6,555 (3·2%) (Supplementary Table 4).

Of the cephalosporins, ceftriaxone accounted for 2,595/9,19 (89%) prescriptions. Cefalexin was the only first-generation cephalosporin used, accounting for 154/2,919 (5%) prescriptions and 4/2,919 (0·1%) patients were prescribed second generation cephalosporins (cefuroxime). Ampicillin was the most prescribed of the penicillin class at 41% (462/1,116) prescriptions, followed by amoxicillin at 35% (407/1,116) and cloxacillin at 19% (212/1,116). The majority of beta lactam/beta-lactamase inhibitors (BL/BLIs) and carbapenem use was in Mahosot Hospital at 67/78 (86%) and 56/57 (98%) prescriptions, respectively. Of BL/BLIs, amoxicillin/clavulanic acid was the most prescribed at 45/78 (58%), followed by ceftriaxone/sulbactam at 30/37 (38%) and cefoperazone/sulbactam at 3/78 (3%) (Supplementary Table 4).

### Prescribing indication

The majority of prescribed antibiotics were broad spectrum^17^ making up 5,163/6,433 (80%) prescriptions. The most common indication for prescribing antimicrobial(s) for patients of all ages was pneumonia in 804 (12%), followed by appendicitis in 397 (6%), sepsis in 304 (5%), skin and soft tissue infection in 270 (4%) and gastrointestinal tract infection in 238 (3·6%). Pneumonia was also the most common indication for prescribing in adults aged ≥15 years and children aged >28 days to ≤5 years at 465/5093 (9%) and 287/730 (39%), respectively. Antimicrobials were prescribed mostly for sepsis in neonates at 172/415 (41%). Tonsillopharyngitis and abdominal infection were more diagnosed more frequently in older children (aged >5 to ≤14 years).

The proportion of patients prescribed antimicrobials varied by age group. Penicillins tended to be used more frequently in infants (aged ≤1 year) at (306/754 (41%)), compared to 109/391 (28%) children aged >1 to ≤5 years and only 55/317 (17%) children aged >5 to ≤14 years (*p <* 0·0001). Overall, cephalosporins, penicillins and aminoglycosides were prescribed more frequently in children aged >5 to ≤14 years at 2,590/5,410 (48%), than children aged ≤5 years at 329/1,145 (29%), *p <* 0·0001. Cephalosporin prescriptions were predominantly in older children (>5 to ≤15 years) and adults (≥15 years), accounting for 2,590/5,410 (48%) prescriptions, compared with 329/1,145 (29%) prescriptions in children ≤5 years (*p <* 0·0001). Antimalarials, antivirals and lincosamides were used in adults only (Supplementary Figure 2).

Among patients who were diagnosed with pneumonia, cephalosporin use was more common in adults (≥15 years) at 254 (55%) than children aged ≤28 days, >28 days to ≤1 year, aged >1 to ≤5 years and >5 to ≤14 years at 5 (15%), 49 (27%), 51 (49%) and 10 (50%), respectively (*p <* 0·0001). Aminoglycosides and penicillins were prescribed more in children than adults (*p <* 0·0001) for pneumonia. A similar pattern was observed in patients with sepsis or skin and soft tissue infection (Table 2).

**Table 2:** Antimicrobial use for the top five treatment indications in patients of all ages in six survey hospitals in Laos from 2017 to 2020.

Indication	Patient age	Any antimicrobials; n (%)	Aminoglyco-sides; n (%)	BL/BLIs; n (%)	Carbapen-ems; n (%)	Cephalos-porin; n (%)	Lincosam-ides; n (%)	Macrolides; n (%)	Nitroimid-azoles; n (%)	Penicill-ins; n (%)	Quinol-ones; n (%)	TMP-SMX; n (%)	Tetracycl-ines; n (%)
Pneumonia (n=804)	≤ 28 days	34 (8)	12 (35)	0	1 (3)	5 (15)	0	0	0	16 (47)	0	0	0
	>28 days to ≤ 1 year	182 (54)	56 (31)	2 (1)	0	49 (27)	0	6 (3)	0	69 (38)	0	0	0
	>1 to ≤5 years	105 (27)	21 (20)	0	0	51 (49)	0	4 (4)	0	29 (28)	0	0	0
	>5 to ≤14 years	18 (6)	1 (6)	0	1 (6)	10 (56)	0	0	1 (6)	4 922)	0	1 (6)	0
	≥15 years	465 (9)	12 (3)	3 (1)	6 (1)	254 (55)	0	120 (26)	20 (4)	39 (8)	8 (2)	0	2 (0·4)
	p-value	<0·0001	<0·0001			<0·0001		<0·0001	0·002	<0·0001			
Appendicitis (n=397)	≤ 28 days	0	0	0	0	0	0	0	0	0	0	0	0
	>28 days to ≤ 1 year	0	0	0	0	0	0	0	0	0	0	0	0
	>1 to ≤5 years	1 (0.3)	0	0	0	1 (100)	0	0	0	0	0	0	0
	>5 to ≤14 years	48 (15)	2 (4)	0	0	27 (56)	0	0	19 (40)	0	0	0	0
	≥15 years	348 (7)	19 (5)	2 (0·6)	2 (0·6)	183 (53)	0	0	140 (40)	1 (0·3)	0	1 (0·3)	0
	p-value	<0·0001	1			0·81			1				
Sepsis (n=303)	≤ 28 days	172 (41)	57 (33)	0	2 (1)	31 (18)	0	2 (1)	5 (3)	74 (43)	1 (0.6)	0	0
	>28 days to ≤ 1 year	13 (4)	5 (38)	0	0	3 (23)	0	0	0	4 (31)	1 (8)	0	0
	>1 to ≤5 years	9 (2)	3 (33)	0	0	3 (33)	0	0	0	3 (33)	0	0	0
	>5 to ≤14 years	14 (4)	0	0	0	10 (71)	0	0	0	4 (29)	0	0	0
	≥15 years	96 (2)	2 (2)	0	8 (8)	60 (63)	0	0	10 (10)	7 (7)	5 (5)	1(1)	2 (2)
	p-value	<0·0001	<0·0001			<0·0001				<0·0001			
SST infection (n=270)	≤ 28 days	15 (4)	6 (40)	0	0	1 (7)	0	0	0	8 (53)	1	0	0
	>28 days to ≤ 1 year	13 (4)	5 (38)	0	0	1 (8)	0	0	0	7 (54)	1	0	0
	>1 to ≤5 years	19 (5)	1 (5)	1 (5)	0	3 (16)	0	0	2 (11)	12 (63)	1	0	0
	>5 to ≤14 years	18 (6)	4 (22)	0	0	3 (17)	0	0	4 (22)	7 (39)	1	0	0
	≥15 years	205 (4)	22 (11)	4 (2)	3 (1)	70 (34)	1 (0·5)	3 (1)	60 (29)	40 (20)	2 (1)	0	0
	p-value	*P =* 0·6	0·001			0·02			0·004	<0·0001			
GI tract infection (excluded appendicitis) (n=238)	≤ 28 days	0	0	0	0	0	0	0	0	0	0	0	0
	>28 days to ≤ 1 year	2 (0·6)	0	0	0	2 (100)	0	0	0	0	0	0	0
	>1 to ≤5 years	0	0	0	0	0	0	0	0	0	0	0	0
	>5 to ≤14 years	6 (2)	1 (17)	0	0	2 (33)	0	0	1 (17)	1 (17)	0	0	0
	≥15 years	230 (4)	1 (0·4)	2 (1)	2 (1)	104 (45)	0	5 (2)	85 (37)	25 (11)	6 (3)	0	0
	p-value	<0·0001				0·31							

**Note**: BL/BLIs: beta-lactam/beta-lactamase inhibitors; TMP-SMX: trimethoprim-sulfamethoxazole; SST: skin and soft tissue; GI: gastrointestinal.

Just over half (3,464/6,555 (53%)) of all prescriptions were for treating infections, while 2,310/6,555 (35%) were for either medical prophylaxis (886/6,555 (13%)) or surgical prophylaxis (1,424/6,555 (22%)) (Table 1). During the 4-year survey period, duration of all surgical prophylaxis was longer than one day. Among surgical prophylaxis prescriptions, 777/1,424 (55%) were for cephalosporins (720/777 (93%) were ceftriaxone), with metronidazole the next most frequent at 345/1,424 (24%), and gentamicin at 192/1,424 (13%). Penicillins (442/886 (50%)) were the most frequently prescribed class for medical prophylaxis, of which 215/442 (49%) were for ampicillin, 214/442 (48%) amoxicillin, and 13/442 (3%) cloxacillin (Supplementary Figure 3). The targeted organ systems of antimicrobial prescriptions varied by age. In infants (aged ≤1 year), antimicrobial prescriptions targeted the respiratory system in 230/754 (31%). In older children and adults, prescriptions were most commonly for the gastrointestinal system at 103/317 (32%) and 1,459/5,093 (29%), respectively (Supplementary Figure 4). Targeted therapy based on microbiology results was documented for 75/4,377 (2%) patients or 88/6,555 (1%) prescriptions. Culture results were only available for 58/75 (77%) patients (*Burkholderia pseudomallei* infection for 28/58 (48%), followed by *Escherichia coli* infection for 8/58 (14%), and *Staphylococcus aureus* infection for 7/58 (12%)). Of 75 patients with confirmed infection, tuberculosis was diagnosed in 17 (23%) patients by either microscopy or GeneXpert (Supplementary Table 5). Among survey hospitals, confirmed diagnoses based on laboratory results were mostly (52/75 (69%) from Mahosot Hospital patients.

### Assessment of appropriate use of antimicrobials

**Figure 3:**
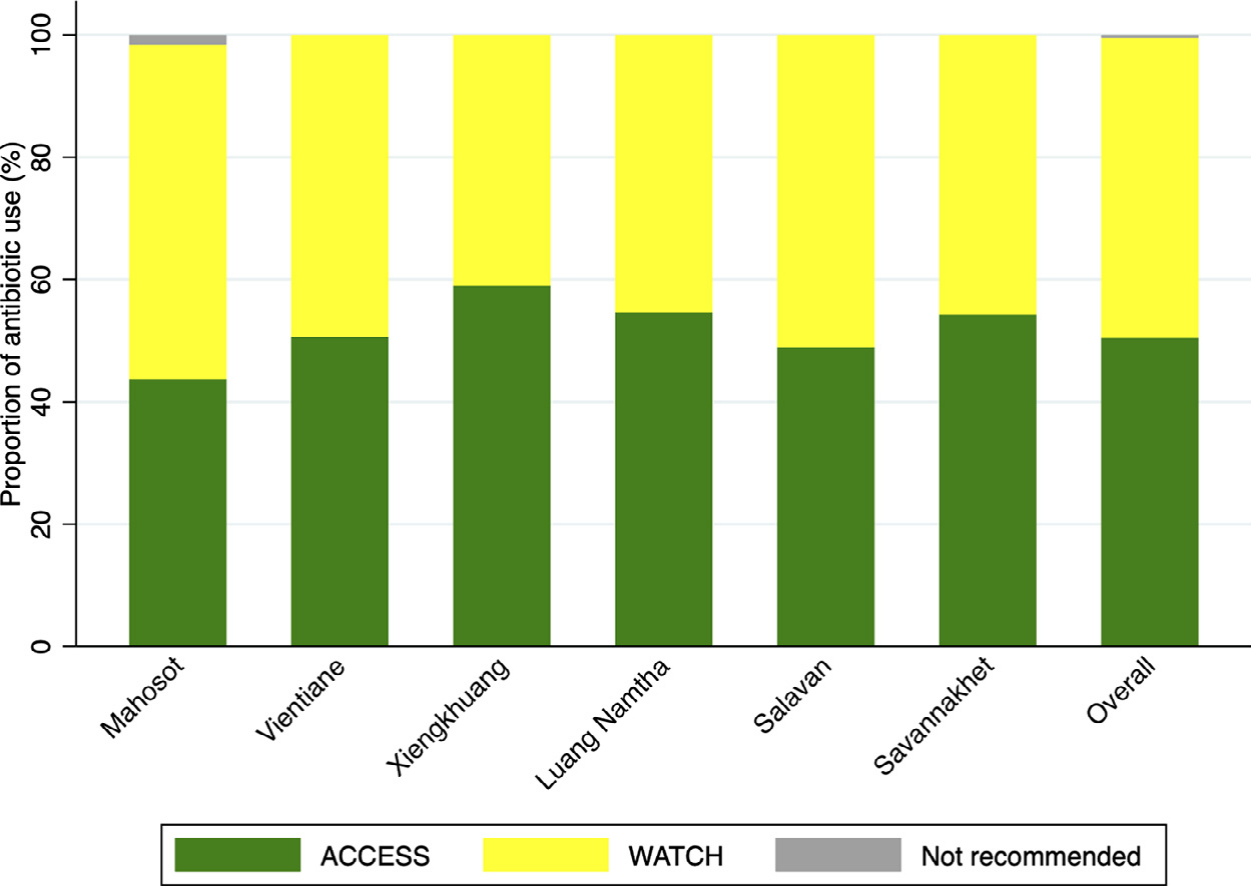
Overall use of antibiotics of different AWaRE classifications in six survey hospitals in Laos, from 2017 to 2020.

Among 4,255 (97%) patients (6,433 (98%) prescriptions) who received antibiotics, the use of ACCESS antibiotics over these four years made up 3,244/6,433 (50%) prescriptions, while WATCH antibiotics accounted for 3,156/6,433 (49%). No RESERVE group antibiotic prescription was captured in this study. However, antibiotics from the ‘Not recommended” group of WHO AWaRe were prescribed to 33/4,255 (0·8%) patients, including ceftriaxone-sulbactam in 30/33 (91%) and cefoperazone-sulbactam in 3/33 (9%). Among the WATCH group used, 44/3,156 (1%) were based on microbiology results while no prescriptions of antibiotics in the ‘Not recommended’ group were suggested by microbiology results. ACCESS antibiotics were more commonly used in children (63%) than adults (47%) (*p <* 0·0001). ACCESS group use was highest in infants aged ≤1 year at 77% (578/754) and 52% (199/384) in children aged between >1 and ≤5 years, 46% (141/309) in children aged between >5 and ≤14 years, and 47% (2,326/4,986) in adults (Supplementary Figure 5). Use of ACCESS group antibiotics varied by hospitals in each survey and ranged from 44%-59% prescriptions. Use of WHO AWaRE ‘Not recommended’ antibiotics was only found in Mahosot Hospital in 33/2,029 (2%) prescriptions (Figure 3).

### Guideline compliance

Of 6,555 prescriptions, 4,397 (67%) could be assessed as to whether the prescriptions adhered to the pre-2021 guidelines, whilst, 2,158 (33%) were unable to be assessed because there was no guidelines or prescribing indications were unknown/unclear. During this 4-year monitoring period, 26% (1,136/4,397) adhered to pre-2021 guidelines and nearly three-quarters of prescriptions (74% (3,261/4,397)) did not adhere. Retrospective analysis of the hypothetical compliance with the new 2021 antimicrobial prescribing guidelines (2021-guidelines)^18^^,^^19^ was performed, with the percentage of antimicrobial prescriptions adherent to the guidelines increasing from 26% (1,136/4,397) for the pre-2021 guidelines to 38% (1,964/5,129) for the 2021-guidelines. At the same time the percentage of prescriptions that could not be assessed decreased from 33% (2,158/6,555) to 22% (1,426/6,555) and the percentage that were not adherent to guidelines decreased from 74% (3,261/4,397) to 62% (3,165/5,129). More details on antimicrobial use in relation to pre-2021 guidelines *versus* 2021-guidelines are in supplementary table 6.

Based on pre-2021 guidelines, adherence to the guidelines was more common among children aged ≤5 years at 44% (450/1,024) than for older children aged >5 to ≤14 years and adults at 20% (686/3,373). Prescriptions not adherent to the guidelines were found most commonly in obstetric/gynaecology departments at 98% (637/648), followed by surgery departments at 81% (1,011/1,248). The five most common indications for prescriptions without guidelines to assess against were eye, cardiovascular system, bone and joints, male genital conditions, and skin and soft tissue problems at 97% (33/34), 94% (33/35), 90% (162/180), 89% (81/91) and 76% (407/535), respectively. The top five indications for prescriptions without guidelines to assess, based on 2021-guidelines, were eye, male genital conditions, cardiovascular system, central nervous system and skin and soft tissue at 94% (32/34), 81% (81/91), 51% (18/35), 46% (80/174) and 38% (202/535), respectively.

Pneumonia was the most common indication identified for antimicrobial prescription in both children and adults; the proportion of pre-2021 guideline adherence in children for which treatment guidelines were available was high, at 77% (166/216) in those aged ≤1 year, 72% (76/105) in children aged >1 to ≤5 years and 56% (10/18) in children aged >5 to ≤14 years but it was much lower in adults at 6% (26/465) (*p* < 0·0001). The proportions of prescriptions adhering to the pre-2021 guidelines were 51% (157/304) for sepsis, 69% (48/70) for meningoencephalitis, 64% (35/55) for suspected typhoid fever, 86% (67/78) for suspected rickettsial infections, and 78% (52/67) for melioidosis (both suspected and confirmed). Of 29 patients (all ages) with microbiologically confirmed melioidosis, antimicrobial prescriptions for two (7%) patients did not adhere to the guidelines.

## Discussion

These first assessments of AMU in Laos found that almost three-quarter of hospitalised patients were receiving antimicrobial treatment on the day of the survey. Third generation cephalosporins (42%), penicillins (17%), nitroimidazoles (16·8%), aminoglycosides (10·4%) and macrolides (3·2%) were the most commonly used antimicrobials. AMU prevalence varied across hospitals over time, and was as high as 88·4% in some hospitals on occasions. This is an alarming message for Laos. This prevalence is higher than reported from other regions of the globe. Our results show higher AMU than the overall proportion from the Global-PPS of 303 hospitals in 53 countries at 34%, in which the highest proportion was from African countries at 50%. The proportion of antimicrobial use in six East and South Asian countries (29 hospitals) was 37% and 42% in west and central Asian countries (27 hospitals in nine countries).^8^ In neighboring Thailand the proportion of hospital antimicrobial use in 183 hospitals in 2018 was 51·5% among 23,686 inpatients^21^ and in Vietnam was 67·4% of 7,571 inpatients from 36 general hospitals in 2008.^13^ Data from a single survey in two hospitals in Yangon, Myanmar, in 2019 showed that antimicrobial use in inpatients was 63·4% (1,255/1,980).^22^ In six hospitals in Indonesia in 2019, 62% of 1,602 inpatients received antimicrobial(s) during the survey period.^23^ The high prevalence of antimicrobial use in Laos was also similar to what has been documented in some low- and middle-income African countries recently, for example Botswana reporting AMU in 70·6% of 711 patients in one survey round in 10 hospitals,^24^ Uganda 74% of 1,077 patients in one survey round from 13 hospitals,^25^ and Nigeria 80·1% of 321 patients in one survey round in three hospitals.^26^

Choice of antimicrobial use varies by country. The prescription patterns in Laos resemble other low- and middle-income countries,^9^^,^^13^^,^^23^^,^^27^^,^^28^ whilst surveys in a high income country such as the USA, western and northern Europe showed that BL/BLIs (including, amoxicillin/clavulanic acid, ampicillin/sulbactam, piperacillin/tazobactam) were most commonly prescribed, followed by 3^rd^ or 4^th^ generation cephalosporins, fluoroquinolones or glycopeptides.^8^^,^^29^ A recent AMR and AMU situational analysis in Laos showed that the proportion of extended-spectrum beta lactamase (ESBL)-producing *Escherichia coli* among cultured isolates of *E.coli* is increasing year on year^14^ (47% (42/90) from urine and 51% (56/109) from blood in 2020).^30^ Frequent use of cephalosporins may be contributing to increasing numbers of ESBL-producing *E. coli* in Laos. With rising levels of antibiotic resistance in Laos for some critical pathogens,^14^ choice of empirical antibiotic treatment must be carefully considered to ensure that patients receive the most appropriate treatment.

The proportion of antimicrobial use adherent to the local pre-2021 treatment guidelines in this study was 26%, while 33% of prescriptions could not be evaluated because of missing guidelines or unknown/unclear indications. Comparison with adherence to 2021-guidelines found an increase in guidelines adherence to 35% and the proportion of use which could not be assessed reduced from 25% to 14%. This is much lower than estimates of appropriate antimicrobial use from other Asian countries (Vietnam (69·2%) in 2008, Myanmar (31·9%) in 2019, Indonesia (52·2%) in 2019 and India (77·4%) in 2017).^13^^,^^22^^,^^23^^,^^27^ Globally, the appropriateness of antimicrobial prescriptions based on local guidelines has been shown to range from 64·1% (Latin America) to 85·8% (North America).^8^ This suggests that a key intervention to improve the appropriate use of antimicrobials in hospitals are comprehensive locally-informed antimicrobial use guidelines which cover all infectious diseases, that are regularly updated, and follow both international recommendations and local antimicrobial resistance patterns. The newly released 2021-guidelines,^18^^,^^19^ and systematic monitoring and feedback on AMU in hospitals in Laos (https://clinicaltrials.gov/ct2/show/NCT04914793), will hopefully lead to increased appropriate use of antimicrobials in Lao hospitals over time. Existing evidence has suggested that guidelines can improve the quality of antimicrobial prescribing. However other factors such as education, policy and public engagement also play important roles in sustainably improving the appropriateness of antimicrobial use.^31^, ^32^, ^33^

Inappropriate surgical prophylaxis (99%) accounted for most inappropriate use. The latest local surgical prophylaxis guidelines were released in 1996 and have not been updated. Antibiotics of choice in the 1996 guidelines were mainly ampicillin (intravenous), cefalotin (intravenous), ± gentamicin or metronidazole; while our current study showed that the majority of the antimicrobial prescriptions for surgical prophylaxis were for ceftriaxone at 50%, whilst ampicillin accounted for 1% of prescriptions and no cefalotin prescriptions were captured. Cefazolin was commonly used in Oceania, North America, Western Europe for surgical prophylaxis, whilst ceftriaxone was used more often in Eastern and Southern Europe, Africa,^8^ Indonesia^23^ and, as shown here, in Laos. Improving rational use of surgical antibiotic prophylaxis is challenging in low- and middle-income countries. Appropriate approaches such as comprehensive guidelines, monitoring of the use, feedback and improving/updating physician's knowledge should be considered. ^34^

The WHO AWaRe list is being used increasingly in many countries to monitor the rational use of antibiotics.^9^^,^^23^^,^^25^^,^^35^, ^36^, ^37^ The overall use of the ACCESS group in this study was 50%, which did not reach the WHO target recommendation (at least 60%).^35^ However, the use of the ACCESS group in infants and children reached 76·6% and 63%, respectively; which was slightly higher than equivalent data from 56 countries, which ranged from 7·8% to 61·2%.^36^ The use of the ACCESS group antibiotics in adult patients in Laos was 46·7%, similar to data from Global-PPS from 69 countries in which the use of ACCESS group ranged from 24·8% to 57·7% in adult patients.^37^ In Laos, 88% of the use of the ‘Not recommended’ group was in surgery departments and 83% was for urinary tract surgical prophylaxis. Antibiotics from this group are not included in the list of essential medicines of Laos.^38^ International recommendations for urologic surgical prophylaxis are fluoroquinolones or first or second generation cephalosporins, or aminoglycosides. Broad-spectrum antibiotics are usually reserved for severe infections.^39^

This study has key limitations. The total duration of therapy was not captured because it was a point prevalence survey. There were no Lao guidelines for treatment or prevention of some infections meaning appropriateness could not be assessed in every patient. The assessment of appropriateness of antimicrobial use in this study was solely based on choice of antimicrobial(s), regardless of the dose, duration and route of administration; this could lead to an overestimate of the appropriateness of use. Data indicating antimicrobial use by hospital-acquired or community-acquired infections were not available. As we did not survey outpatient prescriptions, we do not have the complete picture of hospital antimicrobial use in the country.

These results from repeated surveys over 41 months suggest that Laos has a consistently high prevalence of antimicrobial use in hospitals. Inappropriate use of antibiotics was highest by surgical and obstetrics/gynecology specialties. The choice of available antibiotics to prescribe in Laos is limited. Some useful antibiotics (chloramphenicol, clindamycin or amikacin) are available but rarely used. Access to microbiology diagnosis is also limited outside Vientiane City but has been expanding to many provincial hospitals recently with the support of the Lao government and international funders. Antimicrobial stewardship activities to promote the rational use of antimicrobials in hospitals were sporadic over this survey period. As in many low- and middle-income countries,^40^ Laos faces many challenges to its efforts to combat increasing AMR. The baseline data from this study for Laos present a good opportunity to implement appropriate evidence-based targeted antimicrobial stewardship interventions to improve the situation. These could include operational research to understand the reasons for excess use of antimicrobial prophylaxis, tailored antimicrobial stewardship education to health workers, expanding PPS nationwide with regular feedback from/to hospitals, regular updates of antimicrobial use guidelines in electronic format, and improving access to diagnostic microbiology facilities to support appropriate prescription. Reinforcement and empowerment of hospital drug and therapeutics committees and pharmacists to monitor antimicrobial use and suggest interventions to ensure compliance with guidelines would be an important step.

## Contributors

VC, TR, MM, EA and PN conceptualized and designed the study, SK, PP, VP, CP, SS, KD and SB provided data and facilitated data collection, VC, AD and DC collected data, VC and TR performed data curation, VC analysed data and wrote the original draft. All authors reviewed and approved final version of the manuscript. VC is the guarantor.

## Data sharing statement

Data from this study are a part of national antimicrobial surveillance. De-identified data are available on reasonable request to Lao Ministry of Health.

## Editor note

The Lancet Group takes a neutral position with respect to territorial claims in published maps and institutional affiliations.

## Declaration of interests

H Rogier van Doorn is board member of SEDRIC (Surveillance and Epidemiology of Drug Resistant Infections Consortium). All other authors declare no completing interests. The study received a grant from bioMérieux Singapore to start the project. The funders had no role in study design, data collection and analysis, decision to publish, or preparation of the manuscript.

## Appendix Supplementary materials

Image, application 1

Image, application 2

Image, image 1

Image, image 2

Image, image 3

Image, image 4

Image, image 5
