# Supplementary material for: Evaluation of trends in hospital antimicrobial use in the Lao PDR using repeated point-prevalence surveys-evidence to improve treatment guideline use
Source: Lancet Reg Health West Pac. 2022 Jul 9;27:100531. doi: 10.1016/j.lanwpc.2022.100531 (PMC9283659; doi:10.1016/j.lanwpc.2022.100531)
Supplement: Supplementary file 1 [file mmc1.docx]

**Evaluations of trends in hospital antimicrobial use in the Lao PDR using repeated point-prevalence surveys-evidence to improve treatment guideline use**

**Caption for supplementary material**

**Supplementary Table 1**: Classification of prescribing indication.

**Supplementary Table 2**: List of the most recent treatment guidelines.

**Supplementary Table 3**: The use of antimicrobials by season in six survey hospitals from 2017 to 2020.

**Supplementary Table 4**: The use of antimicrobials in six survey hospitals from 10 surveys from 2017 to 2020.

**Supplementary Table 5**: Targeted therapy based on laboratory results from six survey hospitals from 10 surveys from 2017 to 2020.

**Supplementary Table 6**: Appropriate use of antimicrobials (based on pre 2021 guidelines vs 2021 antimicrobial use guidelines) by age, department types and organ involved in six surveyed hospitals from 2017 to 2020.

**Supplementary Figure 1**: Hospital antimicrobial use point prevalence survey sites in Laos.

**Supplementary Figure 2**: The use of antimicrobials by age group in six survey hospitals from 2017 to 2020.

(Note: BL/BLIs= Beta-lactam/Beta-lactamase inhibitors; TMP-SMX= Trimethoprim-sulfamethoxazole)

**Supplementary Figure 3**: Antimicrobial use by type of indication in six survey hospitals from 2017 to 2020.

(Note: BL/BLIs= Beta-lactam/Beta-lactamase inhibitors; MP = medical prophylaxis; TMP-SMX= Trimethoprim-sulfamethoxazole)

**Supplementary Figure 4**: Antimicrobial use for the top organ systems stated as involved for children and adults in six survey hospitals from 2017 to 2020.

(Note: BL/BLIs= Beta-lactam/Beta-lactamase inhibitors; ENT= ear nose and throat; GI= gastrointestinal; NDS= non-defined site; OBGY= obstetrics and gynaecology; RESP= respiratory; SST= skin and soft tissue; TMP-SMX= Trimethoprim-sulfamethoxazole; UT= urinary tract)

**Supplementary Figure 5**: WHO AWaRe group use by age category among hospitalized patients in six survey hospitals from 2017 to 2020.
