# Supplementary material for: Evaluation of trends in hospital antimicrobial use in the Lao PDR using repeated point-prevalence surveys-evidence to improve treatment guideline use
Source: Lancet Reg Health West Pac. 2022 Jul 9;27:100531. doi: 10.1016/j.lanwpc.2022.100531 (PMC9283659; doi:10.1016/j.lanwpc.2022.100531)
Supplement: Supplementary file 2 [file mmc2.docx]

**Evaluation of trends in hospital antimicrobial use in the Lao PDR using repeated point-prevalence surveys-evidence to improve treatment guideline use**

**SUPPLEMENTARY MATERIAL**

# **Supplementary Table 1: Classification of prescribing indication**

| **Indications** | **Details** |
| --- | --- |
| **Treatment of infection** | Treatment of infection |
| **Medical prophylaxis** | Medical prophylaxis-general: gastrointestinal bleeding, liver cancer, prophylaxis for opportunistic infections in HIV patients, malnutrition, neonatal infection prophylaxis (including preterm delivery, low birthweight or birth asphyxia without sign or symptoms of infections) |
|  | Medical prophylaxis-trauma: e.g., head, eye, chest, abdomen, knee, leg, arm trauma (mostly from road traffic crashes) |
|  | Medical prophylaxis-vaginal delivery: normal delivery via vaginal route with or without episiotomy |
| **Surgical prophylaxis** | Antimicrobial(s) given for prophylaxis purposes, e.g, elective surgery |
| **Unclear** | Not enough information to indicate why antimicrobials were prescribed (e.g. pleural effusion or diarrhoea (only) without further information of possible cause) |
| **Unknown** | Not known why antimicrobial(s) was given |

# **Supplementary Table 2: List of the most recent treatment guidelines**

| **Guidelines** | **Edition** | **Year** | **Available language** | **Publisher** | **Available at** |
| --- | --- | --- | --- | --- | --- |
| Protocoles D’Antibioprophylaxie en Chirurgie | - | 1996 | French | Mahosot Hospital | Mahosot Hospital |
| Diagnosis and treatment in district hospital | 2 | 2004 | Lao/English /French | IFMT | IFMT |
| Malaria treatment guidelines for provincial and district hospitals | - | 2011 | English | CMPE, MoH | CMPE, MoH |
| Guidelines for diagnosis and treatment of uncomplicated malaria for Health centres and village health volunteers/workers, Lao PDR | 1 | 2011 | Lao/English | CMPE, MoH | CMPE, MoH |
| Guidelines for clinical management of HIV and Tuberculosis co-infection | - | 2011 | Lao | CMPE, MoH | MoH |
| National Tuberculosis control program-National technical guidelines | 3 | 2014 | Lao/English | MoH | MoH |
| Standard treatment guidelines | 4 | 2012 | Lao | MoH | MoH |
| Mahosot Microbiology Review | 7 | 2013 | Lao/English | LOMWRU | LOMWRU, University of Health Sciences (UHS) Library [http://uhs-elibrary.la/Elibrary.php?&parentID= 0&CatID=11](http://uhs-elibrary.la/Elibrary.php?&parentID=%200&CatID=11) |
| Pocket book of hospital care for children | 4 | 2017 | Lao/English | WHO, MoH | WHO, MoH |
| Lao PDR National Guidelines for the Use of Antiretroviral Therapy in Adults and Children | 5 | 2017 | English | MoH | MoH |
| National Guidelines for the Treatment of Sexually Transmitted Infections | 4 | 2018 | Lao | MoH | MoH |
| Lao Paediatric Antimicrobial Prescribing Guidelines | 1 | 2020 | Lao | DHR, MoH | DHR, MoH |
| Lao Adult Antimicrobial Prescribing Guidelines | 1 | 2020 | Lao | DHR, MoH | DHR, MoH |

**Note**: IFMT= Institut de la Francophonie pour la Médecine Tropicale; CMPE= Center of Malariology, Parasitology, and Entomology; MoH= Ministry of Health; LOMWRU= Lao-Oxford-Mahosot Hospital Wellcome Trust Research Unit; WHO= World Health Organization; DHR= Department of Healthcare and Rehabilitation.

# **Supplementary Table 3: The use of antimicrobials by season in six survey hospitals from 2017 to 2020**

| **Antimicrobial class** | **2017** | **2018** |  |  |  | **2019** |  |  |  | **2020** |  |  |  |
| --- | --- | --- | --- | --- | --- | --- | --- | --- | --- | --- | --- | --- | --- |
|  | **Wet season (n=533)** | **Hot dry season (n=477)** | **Wet season (n=573)** | **Cool dry season (n=468)** | **p-value** | **Hot dry season (n=648)** | **Wet season (n=812)** | **Cool dry season (n=727)** | **p-value** | **Hot dry season (n=637)** | **Wet season (826)** | **Cool dry season (n=854)** | **p-value** |
| Aminoglycosides (n=679) | 62 (11.6) | 54 (11) | 57 (9.9) | 60 (12.8) | 0.34 | 70 (10.8) | 74 (9.1) | 76 (10.4) | 0.52 | 58 (9.1) | 82 (9.9) | 86 (10) | 0.81 |
| Antifungals (n=47) | 7 (1.3) | 2 (0.4) | 8 (1.4) | 1 (0.2) | 0.07 | 5 (0.8) | 6 (0.7) | 6 (0.8) | 1 | 6 (0.9) | 3 (0.4) | 3 (0.3) | 0.23 |
| Anti-helminthics (n=12) | 0 | 0 | 0 | 0 | - | 3 (0.5) | 2 (0.2) | 3 (0.4) | 0.75 | 1 (0.2) | 1 (0.1) | 2 (0.2) | 1 |
| Antimalarials (n=1) | 0 | 0 | 1 (0.2) | 0 | - | 0 | 0 | 0 | - | 0 | 0 | 0 | - |
| Anti-tuberculosis (n=49) | 9 (1.7) | 4 (0.8) | 5 (0.9) | 7 (1.5) | 0.60 | 4 (0.6) | 6 (0.7) | 3 (0.4) | 0.73 | 3 (0.5) | 3 (0.4) | 5 (0.6) | 0.93 |
| Antivirals (n=13) | 0 | 1 (0.2) | 0 | 0 | - | 5 (0.8) | 3 (0.4) | 0 | 0.05 | 0 | 4 (0.5) | 0 | - |
| BL/BLIs (n=78) | 15 (2.8) | 5 (1) | 5 (0.9) | 6 (1.3) | 0.74 | 12 (1.8) | 14 (1.7) | 8 (1.1) | 0.45 | 0 | 9 (1.1) | 4 (0.5) | - |
| Carbapenems (n=57) | 1 (0.2) | 2 (0.4) | 1 (0.2) | 4 (0.8) | 0.27 | 4 (0.6) | 9 (1.1) | 11 (1.5) | 0.28 | 8 (1.3) | 7 (0.8) | 10 (1.2) | 0.72 |
| Cephalosporins (n=2,919) | 216 (40.5) | 198 (41.5) | 243 (42.4) | 194 (41.4) | 0.94 | 282 (43.5) | 370 (45.6) | 323 (44.4) | 0.73 | 304 (47.7) | 400 (48.4) | 389 (45.5) | 0.47 |
| (n=2) | 0 | 0 | 0 | 0 | - | 0 | 0 | 0 | - | 0 | 1 (0.1) | 1 (0.1) | - |
| Macrolides (n=213) | 34 (6.4) | 22 (4.6) | 25 (4.4) | 21 (4.5) | 0.98 | 18 (2.8) | 19 (2.3) | 34 (4.7) | 0.03 | 8 91.3) | 16 (1.9) | 16 (1.9) | 0.58 |
| Nitroimidazoles (n=1,103) | 65 (12.2) | 80 (16.8) | 78 (13.6) | 64 (13.7) | 0.28 | 100 (15.4) | 143 (17.6) | 126 (17.3) | 0.50 | 124 (19.5) | 159 (19.2) | 264 (19.2) | 0.99 |
| Penicillins (1,116) | 87 (16.3) | 90 (18.9) | 110 (19.2) | 94 (20.1) | 0.88 | 124 (19.1) | 133 (16.4) | 114 (15.7) | 0.20 | 102 (16) | 121 (14.6) | 141 (16.5) | 0.56 |
| Quinolones (n=121) | 20 (3.7) | 10 (2.1) | 17 (3) | 9 (1.9) | 0.52 | 14 (2.2) | 9 (1) | 7 (1) | 0.15 | 11 (1.7) | 7 (0.8) | 17 (2) | 0.12 |
| Trimethoprim-sulfamethoxazole (n=51) | 8 (1.5) | 2 (0.4) | 7 (1.2) | 0 | - | 3 (0.5) | 7 (0.9) | 6 (0.8) | 0.67 | 8 (1.3) | 3 (0.4) | 7 (0.8) | 0.14 |
| Tetracyclines (n=94) | 9 (1.7) | 7 (1.5) | 16 (2.8) | 8 (1.7) | 0.29 | 4 (0.6) | 17 (2.1) | 10 (1.4) | 0.05 | 4 (0.6) | 10 (1.2) | 9 (1) | 0.56 |

**Note**: Hot dry season: March to April; cool dry season: November to February; wet season: May-October, p-value: Chi-squared test or fisher’s exact test were used to test for differences in the proportion of antimicrobial use across different seasons

# **Supplementary Table 4: The use of antimicrobials in six survey hospitals from 10 surveys from 2017 to 2020**

| **Class** | **Antimicrobial agents** | **Hospitals** | | | | | | |
| --- | --- | --- | --- | --- | --- | --- | --- | --- |
|  |  | **Mahosot** | **Vientiane Province** | **Luang Namtha** | **Xiengkhuang** | **Salavan** | **Savannakhet** | **Overall** |
|  |  | n= 2,090 (%) | n= 880 (%) | n= 479 (%) | n= 1,010 (%) | n= 962 (%) | n= 1,134 (%) | n= 6,555 (%) |
| **Antifungals** | Amphotericin B | 8 (0·4) | - | 1 (0·2) |  |  | 3 (0·3) | 12 (0·2) |
|  | Fluconazole | 15 (0·7) | - | 1 (0·2) | - | 1 (0·1) | 11 (1) | 28 (0·4) |
|  | Nystatin | 2 (0·1) | 4 (0·5) | - | - | - | 1 (0·09) | 7 (0·1) |
| **Antihelminthics** | Albendazole | 6 (0·3) | 3 (0·3) | - | - | - | - | 9 (0·1) |
|  | Mebendazole | - | - | 1 (0·2) | - | - | - | 1 (0·02) |
|  | Praziquantel | - | 2 (0·2) | - | - | - | - | 2 (0·03) |
| **Antimalarials** | Chloroquine | 1 (0·05) | - | - | - | - | - | 1 (0·02) |
| **Anti-tuberculosis** | Rifampicin, isoniazid | 1 (0·05) | - | - | - | - | 4 (0·4) | 5 (0·08) |
|  | Rifampicin, pyrazinamide and isoniazid | 1 (0·05) | - | - | 3 (0·3) | 1 (0·1) | - | 2 (0·03) |
|  | Rifampicin, pyrazinamide, ethambutol and isoniazid | 22 (1·1) | 1 (0·1) | 4 (0·8) | - | 9 (0·9) | 3 (0·3) | 42 (0·6) |
| **Antivirals** | Aciclovir | 4 (0·2) | 1 (0·1) | - | - | 1 (0·1) | 1 (0·09) | 7 (0·1) |
|  | Antiretrovirals | 1 (0·05) | - | - | - | - | 5 (0·4) | 6 (0·09) |
| **Aminoglycosides** | Amikacin | 7 (0·3) | - | - | - | - | - | 7 (0·1) |
|  | Gentamicin | 218 (10·4) | 48 (5·5) | 66 (13·8) | 135 (13·4) | 84 (8·7) | 121 (10·7) | 672 (10·3) |
| **BL/BLIs** | Amoxicillin/clavulanic acid | 34 (1·6) | 1 (0·1) | 2 (0·4) | 7 (0·7) | 1 (0·1) | 3 (0·3) | 45 (0·7) |
|  | Cefoperazone/Sulbactum | 3 (0·1) | - | - | - | - | - | 3 (0·05) |
|  | Ceftriaxone/Sulbactam | 30 (1·4) | - | - | - | - | - | 30 (0·5) |
| **Carbapenems** | Imipenem | 6 (0·3) | - | - | - | - | - | 6 (0·09) |
|  | Meropenem | 50 (2·4) | 1 (0·1) | - | - | - | - | 51 (0·8) |
| **Cephalosporins** |  |  |  |  |  |  |  |  |
| First generation | Cefalexin | 40 (1·9) | 5 (0·6) | 14 (2·9) | 23 (2·3) | 23 (2·4) | 49 (4·3) | 154 (2·4) |
| Second generation | Cefuroxime | 4 (0·2) | - | - | - | - | - | 4 (0·06) |
| Third generation | Cefixime | 19 (0·9) | - | - | 2 (0·2) | 1 (0·1) | 1 (0·09) | 23 (0·4) |
|  | Cefotaxime | 14 (0·7) | 3 (0·3) | 2 (0·4) | 14 (1·4) | 9 (1) | 13 (1·2) | 55 (0·8) |
|  | Ceftazidime | 41 (2) | 7 (0·8) | 194 (40·5) | 2 (0·2) | 22 (2·3) | 16 (1·4) | 88 (1·3) |
|  | Ceftriaxone | 850 (40·7) | 398 (45·2) | - | 312 (30·9) | 388 (40·3) | 453 (40) | 2595 (39·6) |
| **Lincosamides** | Clindamycin | 1 (0·05) | - | 1 (0·2) | - | - | - | 2 (0·03) |
| **Macrolides** | Azithromycin | 59 (2·8) | 18 (2·1) | 11 (2·3) | 24 (2·4) | 42 (4·4) | 9 (0·8) | 163 (2·5) |
|  | Clarithromycin | 13 (0·6) | - | 2 (0·4) | 16 (1·6) | 2 (0·2) | - | 33 (0·5) |
|  | Erythromycin | 2 (0·1) | 1 (0·1) | - | 11 (1·1) | 1 (0·1) | 1 (0·09) | 16 (0·2) |
|  | Roxithromycin | 1 (0·05) | - | - | - | - | - | 1 (0·02) |
| **Nitroimidazoles** | Metronidazole | 299 (14·3) | 248 (28·2) | 81 (17) | 104 (10·3) | 171 (17·8) | 200 (17·6) | 1103 (16·8) |
| **Penicillins** | Ampicillin | 47(2·3) | 83 (9·4) | 45 (9·4) | 109 (10·8) | 102 (10·6) | 76 (6·7) | 462 (7·1) |
|  | Amoxicillin | 130 (6·2) | 19 92·2) | 36 (7·5) | 104 (10·3) | 15 (1·6) | 103 (9·1) | 407 (6·2) |
|  | Cloxacillin | 59 (2·8) | 10 (1·1) | 7 (1·5) | 70 (6·9) | 49 (5·1) | 17 (1·5) | 212 (3·2) |
|  | Penicillin G | 9 (0·4) | 3 (0·3) |  | 7 (0·7) | - | 8 (0·7) | 27 (0·4) |
|  | Penicillin V | 3 (0·1) | - | 1 (0·2) | 3 (0·3) | - | 1 (0·09) | 8 (0·1) |
| **Quinolones** | Ciprofloxacin | 24 (1·2) | 1 (0·1) | 1 (0·2) | 18 (1·8) | 11 (1·1) | 1 (0·09) | 56 (0·9) |
|  | Levofloxacin | 12 (0·6) | - | - | - | - | 1 (0·09) | 13 (0·2) |
|  | Norfloxacin | 5 (0·2) | - | - | 8 (0·8) | 3 (0·3) | 1 (0·09) | 17 (0·3) |
|  | Ofloxacin | 8 (0·4) | - | 4 (0·8) | 6 (0·6) | 7 (0·7) | 10 (0·9) | 35 (0·5) |
| **TMP-SMX** | Co-trimoxazole | 22 (1·1) | 9 (1) | - | 3 (0·3) | - | 14 (1·2) | 51 (0·8) |
| **Tetracyclines** | Doxycycline | 19 (0·9) | 14 (1·6) | 5 (1) | 29 (2·9) | 19 (2) | 8 (0·7) | 94 (1·4) |

TMP-SMX= Trimethoprim-sulfamethoxazole; BL/BLIs= Beta-lactam/Beta-lactamase inhibitors

# **Supplementary Table 5: Targeted therapy based on laboratory results from six survey hospitals from 10 surveys from 2017 to 2020**

| **Organism** | **Culture** | **Microscopy/**  **GeneXpert** |
| --- | --- | --- |
| *Burkholderia pseudomallei* | 28 |  |
| *Escherichia coli* | 8 |  |
| *Staphylococcus aureus* | 7 |  |
| *Cryptococcus* spp | 5 |  |
| *Klebsiella pneumoniae* | 3 |  |
| *Pseudomonas aeruginosa* | 3 |  |
| *Talaromyces marneffei* | 2 |  |
| *Streptococcus anginosus* | 1 |  |
| Culture positive but the pathogen names were not recorded in patient's notes | 1 |  |
| *Mycobacterium tuberculosis* |  | 17 |
| **Total** | 58 | 17 |

Note: pathogens were isolated from various specimen types

# **Supplementary Table 6: Appropriate use of antimicrobials (based on pre 2021 guidelines vs 2021 antimicrobial use guidelines) by age, department types and organ involved in six surveyed hospitals from 2017 to 2020**

| **Characteristics** | **Pre 2021 treatment guidelines** | | **2021 antimicrobial use guidelines** | |
| --- | --- | --- | --- | --- |
|  | **Compliance with guidelines; 1,136 (26%)** | **Non compliance with guidelines; 3,261 (74%)** | **Compliance with guidelines; 1,964 (38%)** | **Non compliance with guidelines; 3,165 (62%)** |
| **Age category** |  |  |  |  |
| ≤1 | 302/705 (43) | 403/705 (57) | 319/717 (44) | 398/717 (56) |
| >1-≤5 | 148/319 (46) | 171/319 (54) | 159/326 (49) | 167/326 (51) |
| >5-≤14 | 66/220 (30) | 154/220 (70) | 102/220 (46) | 118/220 (54) |
| ≥15 | 620/3,153 (20) | 2,533/3,153 (80) | 1,384/3,866 (36) | 2,482/3,866 (64) |
| **Department type** |  |  |  |  |
| Intensive Care | 136/409 (33) | 273/409 (67) | 154/431 (36) | 277/431 (64) |
| Medicine | 328/1,048 (31) | 720/1,048 (69) | 596/1,120 (53) | 524/1,120 (47) |
| Obstetric/gynaecology | 11/648 (2) | 637/648 (98) | 47/961 (5) | 914/961 (95) |
| Paediatrics | 363/791 (46) | 428/791 (54) | 401/791 (51) | 309/791 (49) |
| Surgery | 237/1,248 (19) | 1,011/1,248 (81) | 647/1,488 (43) | 841/1,488 (57) |
| Other | 61/253 (24) | 192/253 (76) | 119/338 (35) | 219/338 (65) |
| **Targeted sites for prescriptions** |  |  |  |  |
| Bone and Joint | 4/18 (22) | 14/18 (78) | 24/151 (16) | 127/151 (84) |
| Cardiovascular System | 0 | 2/2 (100) | 5/17 (29) | 12/17 (71) |
| Central Nervous System | 62/89 (70) | 27/89 (30) | 63/94 (67) | 31/94 (33) |
| Ear-Nose-Throat | 42/280 (15) | 238/280 (85) | 51/344 (15) | 293/344 (85) |
| Ophthalmology | 1/1 (100) | 0 | 0 | 2/2 (100) |
| Gastrointestinal | 291/1,198 (24) | 907/1,198 (76) | 730/1,190 (61) | 460/1,190 (39) |
| Male genitalia | 0 | 10/10 (100) | 4/10 (40) | 6/10 (60) |
| Obstetrics/Gynaecology | 6/727 (1) | 721/727 (99) | 58/1,019 (6) | 961/1,019 (94) |
| Respiratory tract | 329/918 (36) | 589/918 (64) | 548/932 (59) | 384/932 (41) |
| Sepsis | 181/328 (55) | 147/328 (45) | 181/341 (55) | 153/341 (45) |
| Skin and Soft Tissue | 22/128 (17) | 106/128 (83) | 46/333 (14) | 287/333 (86) |
| Urinary tract | 75/366 (20) | 291/366 (80) | 116/362 (32) | 246/362 (68) |
| Not defined sites | 123/332 (37) | 209/332 (63) | 131/334 (39) | 203/334 (6661 |
| **Indication for antimicrobial use** |  |  |  |  |
| Treatment of infection | 1,082/2,953 (37) | 1,871/2,953 (63) | 1,692/3,152 (54) | 1,460/3,152 (46) |
| Surgical prophylaxis | 14/1,199 (1) | 1,185/1,199 (99) | 218/1,355 (16) | 1,137/1,355 (84) |
| Medical prophylaxis-general | 36/234 (15) | 198/234 (85) | 50/258 (19) | 208/258 (81) |
| Medical prophylaxis-trauma | 4/11 (36) | 7/11 (64) | 4/73 (5) | 69/73 (94) |
| Medical prophylaxis-vaginal delivery | 0 | 0 | 0 | 291/291 (100) |
