# Supplementary figures and images for: Evaluation of trends in hospital antimicrobial use in the Lao PDR using repeated point-prevalence surveys-evidence to improve treatment guideline use
Source: Lancet Reg Health West Pac. 2022 Jul 9;27:100531. doi: 10.1016/j.lanwpc.2022.100531 (PMC9283659; doi:10.1016/j.lanwpc.2022.100531)

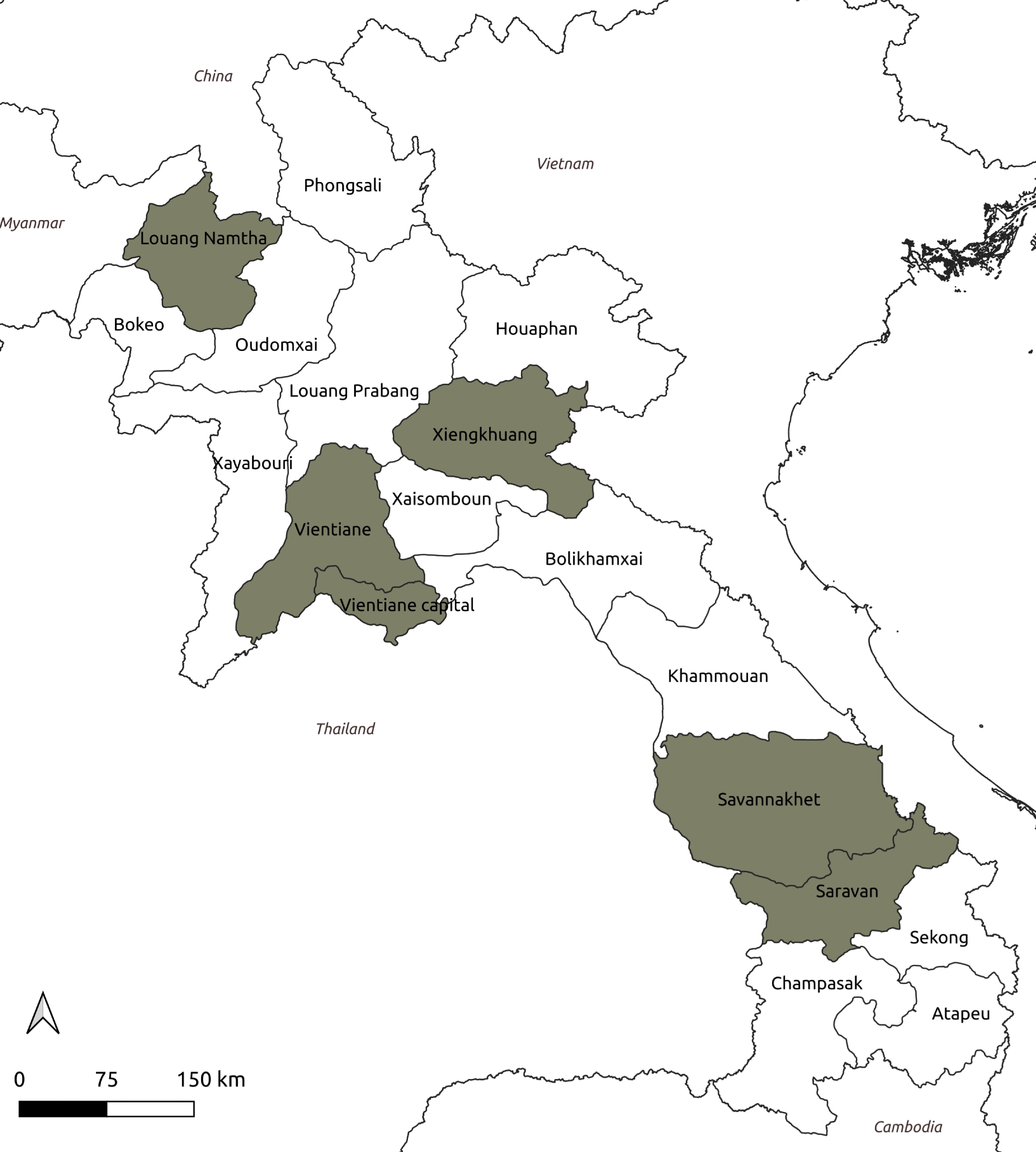

Supplement: Supplementary file 3 [file mmc3.jpg]

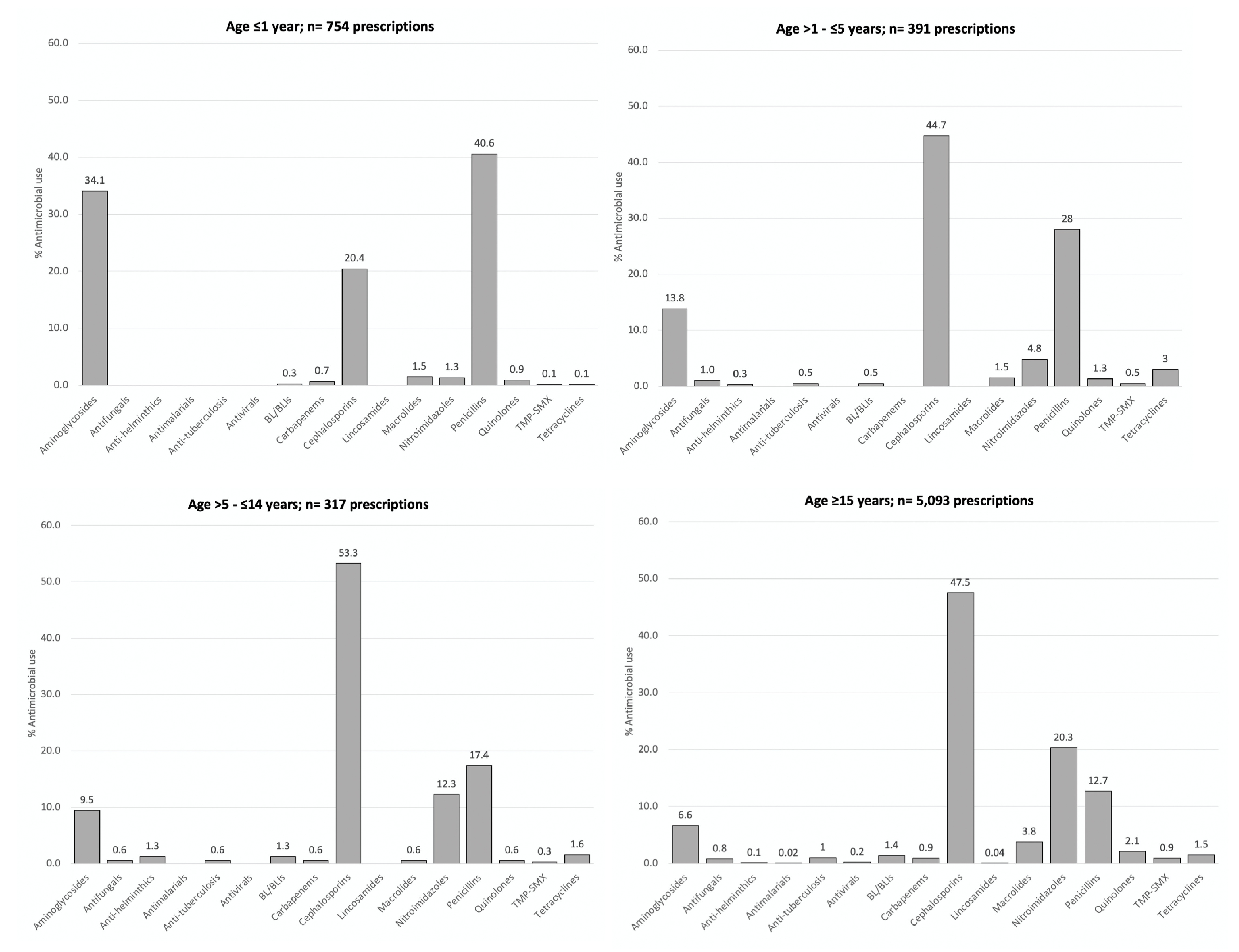

Supplement: Supplementary file 4 [file mmc4.jpg]

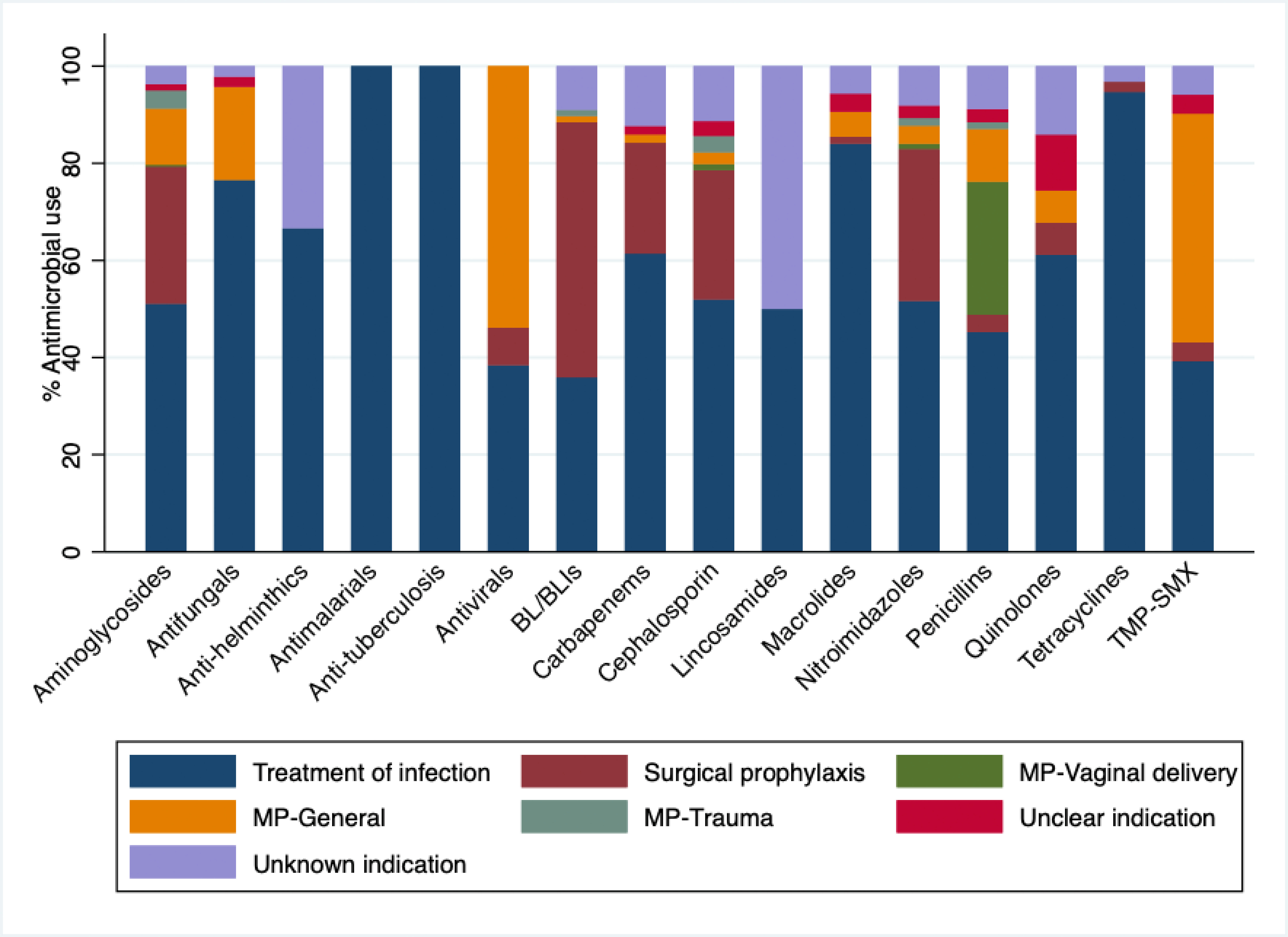

Supplement: Supplementary file 5 [file mmc5.jpg]

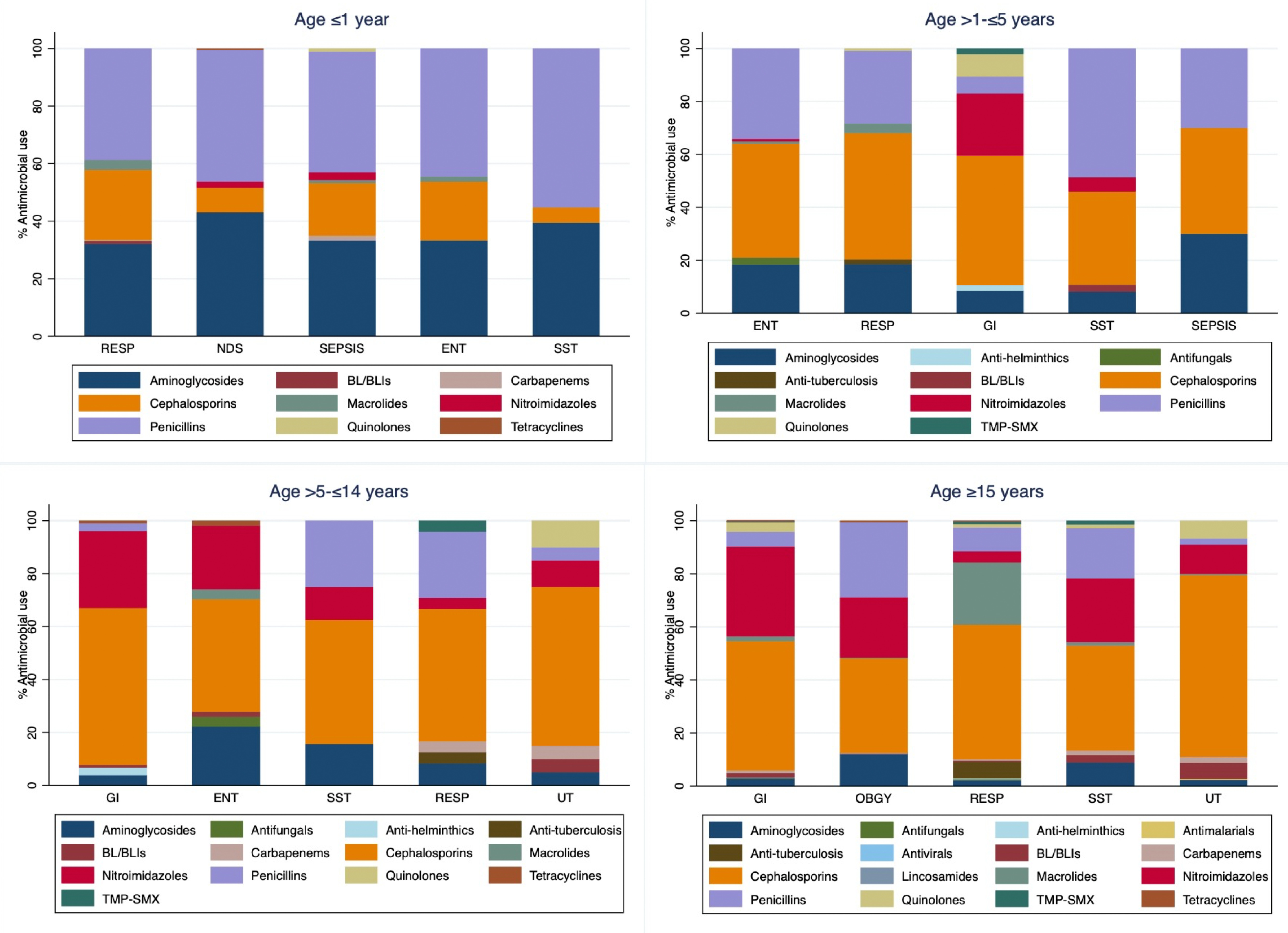

Supplement: Supplementary file 6 [file mmc6.jpg]

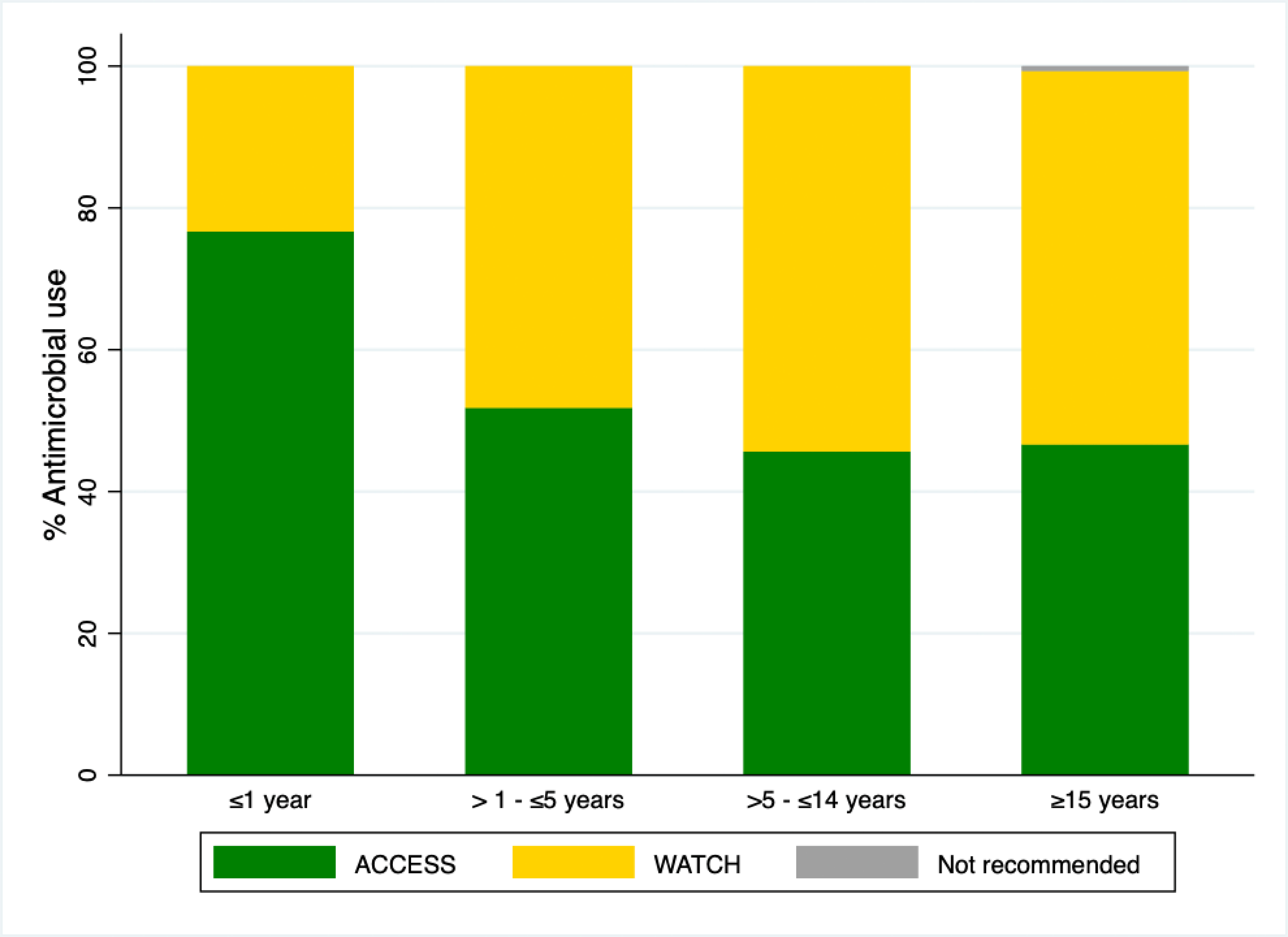

Supplement: Supplementary file 7 [file mmc7.jpg]
